# Voluntary policies on checkout foods and healthfulness of foods displayed at, or near, supermarket checkout areas: a cross-sectional survey

**DOI:** 10.1017/S1368980018002501

**Published:** 2018-10-12

**Authors:** Chi Ching Vivian Lam, Katrine T Ejlerskov, Martin White, Jean Adams

**Affiliations:** 1 Department of Public Health & Primary Care, Institute of Public Health, University of Cambridge, Cambridge, UK; 2 Centre for Diet & Activity Research, MRC Epidemiology Unit, University of Cambridge, Box 285 Institute of Metabolic Science, Cambridge Biomedical Campus, Cambridge CB2 0QQ, UK

**Keywords:** Supermarket, Food retailing, Voluntary commitments, Food marketing

## Abstract

**Objective:**

To determine if voluntary policies on supermarket checkout foods are associated with a difference in the healthfulness of foods displayed at, or near, supermarket checkout areas.

**Design:**

Cross-sectional survey of foods at, or near, supermarket checkouts categorised as less healthy or not according to the Food Standards Agency’s Nutrient Profiling Model.

**Setting:**

One city in Eastern England (population about 125 000).

**Subjects:**

All stores in nine supermarket groups open for business in June–July 2017 in the study city. Supermarket checkout food policies were categorised as clear and consistent, vague or inconsistent, or none.

**Results:**

In thirty-three stores, 11 434 checkout food exposures were recorded, of which 8010 (70·1 %) were less healthy; and 2558 foods in areas near checkouts, of which 1769 (69·2 %) were less healthy. After adjusting for a marker of store size, the odds of a checkout food exposure being ‘less healthy’ was lower in stores with vague or inconsistent checkout policies (OR=0·63; 95 % CI 0·49, 0·80) and in stores with clear and consistent checkout policies (OR=0·33; 95 % CI 0·24, 0·45), compared with no policy. There was no difference in the odds of foods near, but not at, checkouts being less healthy according to checkout food policy.

**Conclusions:**

Supermarket checkout food policies were associated with lower odds of checkout foods but not foods near, but not at, checkouts being less healthy. Further research is required to explore impacts on purchasing and consumption.

Exposure to energy-dense, nutrient-poor foods and drinks contributes to the development of obesity^(^ ^1^ ^)^. One potential source of such foods, which has gained media^(^ ^2^ ^)^, campaign group^(^ ^3^ ^,^ ^4^ ^)^ and research^(^ ^5^ ^–^ ^10^ ^)^ attention, is supermarket checkouts.

Globally, supermarket checkout foods tend to be less healthy and positioned to attract children^(^ ^5^ ^–^ ^9^ ^)^. Foods at checkouts can lead to impulse purchases and child purchasing requests^(^ ^8^ ^,^ ^11^ ^,^ ^12^ ^)^, which parents can find hard to resist^(^ ^4^ ^,^ ^13^ ^)^. The balance of healthier to less healthy checkout foods influences purchasing, with healthier foods being more likely to be selected when they dominate^(^ ^14^ ^)^.

A number of UK supermarkets have policies limiting the display of ‘less healthy’ foods at checkouts. A large scoping review on retail micro-environments in 2013 identified that changing the availability of healthy foods can alter purchasing^(^ ^15^ ^)^; but no studies on checkout foods were identified. Since then, a small number of researcher-led studies have reported mixed effects of changing supermarket checkout foods^(^ ^13^ ^,^ ^16^ ^–^ ^19^ ^)^. These inconsistent results are likely due to variations in the types of products targeted and the level of implementation achieved. The only previous study we are aware of on the impact of supermarket-led checkout food policies found that stores with policies were less likely to display foods at checkouts than stores without policies; and that the foods displayed were more likely to be ‘healthier’^(^ ^20^ ^)^. That study also found that supermarkets adhered well to their checkout food policies, especially if they were clear and consistent.

In the UK, supermarket checkout food policies currently take the form of voluntary commitments. There is substantial scepticism about the potential for such commitments to lead to meaningful public health change^(^ ^21^ ^–^ ^23^ ^)^. This is fuelled in part by evidence that previous voluntary agreements between government and food industry organisations tend to focus on less effective intervention strategies and reflect actions that companies were already doing, or planning to do, at the time agreements were made^(^ ^21^ ^,^ ^24^ ^–^ ^26^ ^)^.

While current UK supermarket checkout food policies are voluntary commitments made by supermarkets, they are not voluntary agreements between food retailers and government^(^ ^27^ ^)^. Instead they are retailer-led, self-regulatory, voluntary actions without any government involvement. Several reasons why industries self-regulate have been described^(^ ^28^ ^)^. In the case of supermarket checkout foods, the most likely is the threat to public relations associated with less healthy checkout foods, following campaigns that have highlighted how difficult shoppers find it to resist child purchasing requests for checkout foods^(^ ^3^ ^,^ ^4^ ^,^ ^13^ ^)^.

Removing less healthy foods from supermarket checkouts may, therefore, improve the customer experience, as well as public health. But if it also leads to decreased sales this could be at odds with supermarkets’ commercial interests. When commercial and public health interests are in conflict, there is an incentive for self-regulatory policies to be weakly conceived and poorly enforced^(^ ^23^ ^,^ ^28^ ^)^. One way in which supermarkets might undermine checkout food policies to avoid commercial impacts is to display less healthy checkout-type foods in areas near, but not at, checkouts. These areas include aisle ends opposite checkouts – another area associated with impulse purchases, with about 40 % of purchases estimated to be made from aisle ends^(^ ^29^ ^)^.

The aim of the current study was to determine if the presence and nature of voluntary policies on supermarket checkout foods were associated with a difference in the healthfulness of foods displayed at, or near, supermarket checkout areas.

## Methods

We conducted a cross-sectional survey in June–July 2017 of foods at, or near, supermarket checkouts in one city in Eastern England with a population of about 125 000. The city is less deprived and more healthy than England as a whole^(^ ^30^ ^)^ and was chosen for pragmatic reasons.

### Supermarket and store selection

We included all stores open for business in June–July 2017 located within the administrative boundaries of the study city and that belonged to one of the nine supermarket groups that together account for more than 90 % of the UK grocery market^(^ ^31^ ^)^. Thirty-two stores were identified via the ‘store locater’ function on supermarket group websites. During data collection, one additional store was identified and included. Two of the nine supermarket groups did not have stores in the study city. As our intention was to study the associations between checkout food policies and foods displayed, rather than ‘name and shame’ particular supermarket groups, we have chosen not to identify specific supermarket groups in the ‘Results’ section of the present paper.

### Foods at, or near, supermarket checkout areas

We defined supermarket checkout areas as any area that customers must pass through to pay for purchases^(^ ^10^ ^,^ ^20^ ^)^. All foods and non-alcoholic drinks (collectively referred to as ‘foods’ hereafter) within the researcher’s arm’s reach (~0·5 m) of these areas were considered foods at checkout areas. We defined areas near to checkout areas as any area within five paces (~3·5 m) of any part of a checkout area that did not meet the definition of checkout areas. Foods within arm’s reach of these areas were considered foods near, but not at, checkouts. Alcoholic drinks were excluded as these are excluded by the policy-relevant tool used to determined ‘healthfulness’ of foods described below.

We used the concept of ‘checkout journeys’ to quantify ‘checkout food exposures’^(^ ^10^ ^,^ ^20^ ^)^. A checkout journey was defined as a route through a checkout area (as defined above). We first determined all possible checkout journeys in each store. We then calculated checkout food exposures as the sum of the number different food lines in each possible checkout journey in each store. In many stores, shared queuing areas leading to multiple payment points make numerous different checkout journeys possible. Any food lines in shared queuing areas were multiple counted to reflect the total number of possible checkout journeys through the shared area. For example, chewing gum displayed in a shared queuing area leading to five payment points was counted five times; whereas chewing gum displayed at a single payment point was counted once.

Foods near, but not at, checkouts were only counted once as the number of possible customer paths past these displays was indeterminable (although likely to be greater than one).

### Data collection

Data collection was conducted by one researcher (C.C.V.L.). The researcher visited all study stores and recorded the number of checkouts in each store, and all food lines displayed at, or near, checkout areas. Only the range of food lines was recorded, not the number of units (or ‘facings’) present. This reflects previous approaches^(^ ^10^ ^,^ ^20^ ^)^, and minimised the intrusiveness of data collection and any disruption to stores. Where the same food line was displayed in a range of package sizes, only the presence of the line was recorded, not the package sizes. Thus, if a particular brand of salt & vinegar potato crisps was displayed in small and large packages, only the presence of that brand and flavour of potato crisps was recorded. Where similar products were present in a number of flavour variants, all variants were recorded. Thus, if both salt & vinegar and cheese & onion flavours of the same brand of potato crisps were displayed, both flavours were recorded.

Data were recorded in-store using a mobile telephone voice recorder. Recordings were downloaded and transcribed within three days of recording. This method has previously been found to have high inter-rater reliability^(^ ^10^ ^)^. To confirm this, a second researcher (E.T.-M.) repeated data collection independently in one study store selected for convenience. There was 95 % agreement between researchers on products recorded.

### Healthfulness of foods at, or near, supermarket checkout areas

All foods identified during data collection were categorised as ‘less healthy’ or ‘healthier’ using the Food Standards Agency’s Nutrient Profiling Model^(^ ^32^ ^)^. This model balances ‘positive’ and ‘negative’ nutrients and food components to arrive at an overall score. Standard cut-offs are used to determine if a food is less healthy. The model is used to determine which foods can be advertised to children on UK television and has reasonable specificity and sensitivity^(^ ^33^ ^)^.

To determine Nutrient Profiling Model scores, nutritional information on observed foods was obtained from one of three sources. We gave preference to information published on manufacturer’s websites, followed by UK supermarket websites, followed by an online crowdsourced database of branded foods (https://www.world.openfoodfacts.org). When no branded data from any of these sources could be found, we used data from an equivalent unbranded product in the Composition of Foods Integrated Dataset^(^ ^34^ ^)^.

### Presence and nature of supermarket checkout food policies

We used data collected in May–September 2017 to determine the presence and nature of supermarket checkout food policies^(^ ^20^ ^)^. We searched the annual reports and webpages of included supermarket groups for information on their checkout food policies and contacted supermarkets’ customer services for further information as needed. When information was not available by these methods, we used information from newspaper articles or other secondary sources.

We categorised checkout food policies into two groups^(^ ^20^ ^)^: clear and consistent policies were those that provided clear information on inappropriate and appropriate checkout foods and applied consistently to all checkouts in stores; vague or inconsistent polices provided vaguer product information or did not apply consistently to all checkouts (or both). One supermarket group had different policies for different store formats (i.e. convenience stores *v*. large hypermarkets). Checkout food policies of included supermarket groups are summarised in Table 1.

**Table 1:** Checkout food policies in seven UK supermarket groups included in the study (adapted from Ejlerskov *et al*.^(^ ^20^ ^)^)

Supermarket group (format)*	Checkout food policy category	Products that should not be displayed at checkouts	Products that can be displayed at checkouts	Applicable checkouts	Information source	Year (month) of implementation
1	Clear & consistent	Sweets & chocolate	Healthier snacks such as dried fruit, nuts and cereal bars; 5-a-day, no ‘red’ traffic light ratings, in calorie-controlled packs, or deemed by Department of Health a ‘healthier snack’	All checkouts	Annual report, supermarket web page	2015 (January)
2 (format a)	Vague or inconsistent	Confectionery	Not stated	Checkouts where families expected to shop with a trolley	Customer service, June 2017	2004 (unknown)
2 (format b)	None	NA	NA	NA	Customer service, June 2017	NA
3	Vague or inconsistent	Confectionery	‘Guilt free’ checkouts (not defined)	1/3 of checkouts	Radio interview and consumer report	2012 (unknown)
4	Clear & consistent	Confectionery, chocolate & sweets	Healthier options including dried fruit, nuts, juices and water	All checkouts	Supermarket web page	2015 (January)
5	None	NA	NA	NA	Customer service, May 2017	NA
6	Vague or inconsistent	Sweets	Not stated	All checkouts	Customer service, September 2017	2014 (August)
7	Vague or inconsistent	Confectionery, crisps, cakes & biscuits	Not stated	All checkouts in company-owned stores, not franchise stores	Customer service, June 2017	2015 (September)

NA, not applicable.

* In one supermarket group, a different checkout food policy was applied to different store formats (e.g. large hypermarket *v*. city centre convenience store).

### Data analysis

We conducted analysis at the store level (*n* 33) with the outcome of interest being the proportion of checkout food exposures or foods near, but not at, checkouts which was less healthy. We used log-binomial regression to determine the odds of a checkout food exposure or food near, but not at, a checkout being less healthy in stores with clear and consistent, or vague or inconsistent checkout food policies, compared with those with no checkout food policy. This approach has been recommended for modelling dependent variables which, as here, are proportions^(^ ^35^ ^)^. Smaller stores may be more cramped making it harder to impose checkout food policies. As such, we adjusted models for the total number of checkouts in each store (a proxy for store size). Standard errors, and hence 95 % CI, were adjusted for clustering of stores at the supermarket group level. There were no stores with no food at checkouts. In stores where there was no food near, but not at, checkouts (*n* 3) these stores were excluded from the relevant analyses.

### Ethics

In line with current guidance, we did not seek ethical approval for the present study which did not include any human, or animal, participants. Store managers were not explicitly asked permission for observations to take place. At no point was the researcher challenged by a member of store staff.

## Results

All thirty-three stores, in seven supermarket groups, identified as meeting the inclusion criteria were included. Food was found at one or more checkouts in all stores, but three stores did not have any food in areas near checkouts.

We identified 11 434 checkout food exposures, of which 8010 (70·1 %) were less healthy; and 2558 foods near, but not at, checkouts, of which 1769 (69·2 %) were less healthy. Table 2 provides information on the number of stores, checkouts, checkout food exposures and foods near, but not at, checkouts by supermarket group and format.

**Table 2:** Proportion of checkout food exposures and foods near checkouts that were less healthy, by checkout food policy category, in a survey conducted in all stores (*n* 33) in seven major UK supermarket groups open for business in one city in Eastern England, June–July 2017

Supermarket group (format)	Checkout food policy category	Stores (*n*)	Checkouts (*n*)	Mean checkouts per store (*n*)	Checkout food exposures					Foods near, but not at, checkouts				
					Less healthy		Healthier		Total	Less healthy		Healthier		Total
					*n*	%	*n*	%	*n*	*n*	%	*n*	%	*n*
2 (format b)	None	4	32	8·0	930	77·1	277	22·9	1207	140	75·3	46	24·7	186
5	None	11	71	6·5	1054	68·7	481	31·3	1535	722	72·0	281	28·0	1003
Sub-total	None	15	103	6·9	1984	72·4	758	27·6	2742	862	72·5	327	27·5	1189
2 (format a)	Vague or inconsistent	2	60	30·0	3323	87·9	456	12·1	3779	87	49·7	88	50·3	175
3	Vague or inconsistent	1	30	30·0	551	64·7	301	35·3	852	0	0	0	0	0
6	Vague or inconsistent	2	42	21·0	364	50·4	358	49·6	722	91	63·6	52	36·4	143
7	Vague or inconsistent	4	40	10·0	668	56·8	508	43·2	1176	327	78·8	88	21·2	415
Sub-total	Vague or inconsistent	9	172	19·1	4906	75·1	1623	24·9	6529	505	68·9	228	31·1	733
1	Clear & consistent	8	80	10	960	54·5	803	45·5	1763	399	63·0	234	37·0	633
4	Clear & consistent	1	10	10	160	40·0	240	60·0	400	3	100	0	0	3
Sub-total	Clear & consistent	9	90	10	1120	51·8	1043	48·3	2163	402	63·2	234	36·8	636
Total	All stores	33	365	11·1	8010	70·1	3424	29·9	11 434	1769	69·2	789	30·8	2558

In stores with no checkout food policy, 72·4 % of checkout food exposures were less healthy, 75·1 % were less healthy in stores with vague or inconsistent policies, and 51·8 % were less healthy in stores with clear and consistent policies (Table 2). After adjusting for the number of checkouts in stores, the odds of a checkout food exposure being less healthy was lower in stores with vague or inconsistent policies compared with no policy (OR=0·63; 95 % CI 0·49, 0·80) and between stores with clear and consistent *v*. no policy (OR=0·33; 95 % CI 0·24, 0·45; Table 3).

**Table 3:** Odds ratios of checkout food exposures and foods near, but not at, checkouts being less healthy, by checkout food policy category, in a survey conducted in all stores (*n* 33) in seven major UK supermarket groups open for business in one city in Eastern England, June–July 2017

		Proportion checkout food exposures less healthy		Proportion foods near, but not at, checkouts less healthy	
Variable	Level	OR*	95 % CI	OR*	95 % CI
Checkout food policy category	None	Reference		Reference	
	Vague or inconsistent	0·63	0·49, 0·80	0·90	0·20, 4·03
	Clear and consistent	0·33	0·24, 0·45	1·21	0·65, 2·23
Checkouts per store (*n*)		1·01	0·99, 1·05	0·97	0·91, 1·02

* Adjusted for clustering at the supermarket group (format) level.

In total, 72·5 % of foods near, but not at, checkouts were less healthy in stores with no checkout food policy, 68·9 % in those with vague or inconsistent policies, and 63·2 % in those with clear and consistent policies (Table 2). After adjustment for the number of checkouts in stores, there was no evidence of a difference in the odds of foods near, but not at, checkouts being less healthy according to checkout food policy type (Table 3).

## Discussion

### Summary of main findings

We conducted a census of foods at, and near, supermarket checkouts in one city in Eastern England. This is the first assessment of foods near, but not at, checkouts we are aware of, and the first to explore associations between difference supermarket checkout policies and these foods. After adjusting for a marker of store size, we found that the proportion of checkout food exposures that were ‘less healthy’ was lowest in stores with clear and consistent checkout food policies, intermediate in stores with vague or inconsistent policies and highest in stores with no policy. There was no difference in the proportion of less healthy foods near, but not at, checkouts according to checkout food policy.

### Strengths and limitations of methods

We conducted a census of all supermarkets in one city, eliminating any internal sampling bias. However, the study city is not representative of the UK in terms of deprivation and health status of residents, or the supermarket groups present. This may limit external validity. As it is unlikely that the impact of checkout food policies on foods at, or near, checkouts varies between cities, the findings should be generalizable across the seven supermarket groups included. Together these account for around 80 % of the UK grocery market^(^ ^31^ ^)^.

We made substantial attempts to collect accurate data on supermarkets’ checkout food policies. However, in some cases we were forced to rely on secondary sources. This may introduce error. The data collection method has previously been reported to have high inter-rater reliability^(^ ^10^ ^)^ and we confirmed this.

There are likely to be seasonal variations in foods displayed at, and near, checkouts. There may also be seasonal variations in the impact of supermarket checkout food policies on what foods are displayed. For example, if supermarkets place particular emphasis on particular seasonal promotions, these may override checkout food policies at some times of the year.

We used the Food Standards Agency’s Nutrient Profiling Model to classify foods as ‘less healthy’ or ‘healthier’^(^ ^32^ ^)^. This is policy-relevant to the UK context. While objective, it does not necessarily reflect the intention of supermarket checkout food policies, or the full spectrum of healthfulness of foods. Nor does it include alcohol.

Our data reflect what foods are displayed, not necessarily what customers buy or consume. No account was taken of the number of ‘facings’ (or units) of different products displayed or how much shelf space each product accounted for. This may also influence purchasing and consumption. Further research is required to explore the impact of checkout food policies on food purchasing and consumption. While we weighted foods displayed at checkouts according to an indicator of customer exposure, there was no comparable way to do the same for foods in areas near checkouts.

Our analyses focus on the proportion of foods at, or near, checkouts that was less healthy. This does not take into account that there were no foods at some checkouts and in some stores that there were no foods near, but not at, checkouts. It is possible that the absence of foods in these areas represents the ‘healthiest’ condition.

### Comparison of findings with previous studies

Across all stores, we found that 70 % of checkout food exposures were for less healthy foods. This is comparable to previous findings from supermarkets and non-food stores in the UK which revealed about 70–80 % of checkout foods to be less healthy^(^ ^5^ ^,^ ^10^ ^)^. At least 70 % of supermarket checkouts in Australia, Canada, Denmark, New Zealand, Sweden, the UK and the USA displayed at least one of confectionery, crisps, chocolate and soft drinks^(^ ^6^ ^,^ ^8^ ^,^ ^9^ ^)^.

In line with previous research^(^ ^20^ ^)^, we found some suggestion of a trend in the proportion of checkout foods that were less healthy from stores with clear and consistent, through vague or inconsistent, to no policies.

### Interpretation and implications of findings

Overall, we found that a high proportion of foods at, and near, supermarket checkouts was ‘less healthy’ and would not be permitted to be advertised to children on UK television. This indicates a substantial public health concern.

However, the proportion of checkout food exposures that were less healthy was substantially lower in stores with checkout food policies than in those with no policy. This indicates that it may be possible to reduce the proportion of less healthy supermarket checkout food exposures, and that alternative foods appear to be available. However, our data are cross-sectional and it is not necessarily the case that it is the checkout food policies that are responsible for the differences seen. For example, supermarkets with healthier checkout foods originally may have been more likely to implement checkout food policies. It has been proposed that there should be government regulation on checkout foods, although no details have yet been developed^(^ ^3^ ^,^ ^36^ ^)^.

In addition to finding a lower proportion of less healthy checkout foods in stores with checkout food policies *v*. no policies, we found no difference in the proportion of less healthy foods near, but not at, checkouts across different checkout food policy groups. Thus, it appears that stores with policies are not undermining their checkout food policies by placing greater proportions of less healthy foods near checkouts to make up for any reductions in sales associated with removing them from checkouts. In contrast, previous research has found little evidence of public health gain from voluntary agreements between government and the food industry^(^ ^21^ ^,^ ^27^ ^)^ or from food industry self-regulation^(^ ^22^ ^,^ ^28^ ^,^ ^37^ ^–^ ^39^ ^)^ for public health benefit. One potential reason for this divergence is that checkout food policies are conceived by supermarkets as enhancing customers’ shopping experience, rather than as a public health measure^(^ ^13^ ^)^. These policies thus converge, rather than conflict, with supermarkets’ commercial interests and there is no incentive for supermarkets to undermine them^(^ ^13^ ^,^ ^28^ ^)^. Qualitative work exploring why supermarket groups adopt the checkout food policies they do may offer further insights for maximising the potential of retail self-regulation for public health gain. Further focus on ‘win–win’ policies with commercial as well as public health benefits may be fruitful, although it is not clear how common these are^(^ ^23^ ^)^.

As we did not analyse purchasing or consumption data, we cannot draw definitive conclusions on the public health impact of checkout food policies. Stores with fewer less healthy checkout food exposures may make up for this by aggressively marketing checkout-type foods elsewhere in store. Future research is required to explore these issues.

## Conclusions

In a survey of all thirty-three branches of large UK supermarket retailers in one English city, we found that the proportion of less healthy checkout foods was lower in stores with checkout food policies. However, there was no difference in the proportion of foods near, but not at, checkouts that was less healthy by checkout food policy. Further research is required to explore impacts on purchasing and consumption. All supermarket checkout food policies in the UK are voluntarily developed and adopted by retailers. Further research is required to determine why the presence of these self-regulatory efforts is associated with greater differences in outcomes than some other self-regulatory efforts to improve public health. Framing self-regulation with potential for public health gain in terms of commercial benefit may be one way of maximising the impacts of this approach.
